# Loss of EGFR signaling-regulated miR-203 promotes prostate cancer bone metastasis and tyrosine kinase inhibitors resistance

**DOI:** 10.18632/oncotarget.1994

**Published:** 2014-05-20

**Authors:** Man Kit Siu, Wassim Abou-Kheir, Juan Juan Yin, Yung-Sheng Chang, Ben Barrett, Florent Suau, Orla Casey, Wei-Yu Chen, Lei Fang, Paul Hynes, Yao-Yu Hsieh, Yen-Nien Liu, Jiaoti Huang, Kathleen Kelly

**Affiliations:** ^1^ Graduate Institute of Cancer Biology and Drug Discovery, College of Medical Science and Technology, Taipei Medical University, Taipei, Taiwan; ^2^ Department of Anesthesiology, Wan Fang Hospital, Taipei Medical University, Taipei, Taiwan; ^3^ Department of Anatomy, Cell Biology and Physiological Sciences Faculty of Medicine, American University of Beirut, Beirut, Lebanon; ^4^ Cell and Cancer Biology Branch, National Cancer Institute, National Institutes of Health, Bethesda, MD, USA; ^5^ Department of Pathology, Wan Fang Hospital, College of Medicine, Taipei Medical University, Taipei, Taiwan; ^6^ Division of Hematology and Oncology, Shuang Ho Hospital, Taipei Medical University, New Taipei City, Taiwan; ^7^ Center of Excellence for Cancer Research, Wan Fang Hospital, College of Medicine, Taipei Medical University, Taipei, Taiwan; ^8^ Department of Pathology and Laboratory Medicine, University of California, Los Angeles, CA, USA

**Keywords:** Bone metastasis/Epidermal growth factor receptor (EGFR)/Prostate cancer/miR-203/KRAS/Tyrosine kinase inhibitors (TKIs) resistance

## Abstract

Activation of EGFR signaling pathway leads to prostate cancer bone metastasis; however, therapies targeting EGFR have demonstrated limited effectiveness and led to drug resistance. miR-203 levels are down-regulated in clinical samples of primary prostate cancer and further reduced in metastatic prostate cancer. Here we show that ectopic miR-203 expression displayed reduced bone metastasis and induced sensitivity to tyrosine kinase inhibitors (TKIs) treatment in a xenograft model. Our results demonstrate that the induction of bone metastasis and TKI resistance require miR-203 down-regulation, activation of the EGFR pathway via altered expression of EGFR ligands (*EREG* and *TGFA*) and anti-apoptotic proteins (API5, BIRC2, and TRIAP1). Importantly, a sufficient reconstitution of invasiveness and resistance to TKIs treatment was observed in cells transfected with anti-miR-203. In prostate cancer patients, our data showed that miR-203 levels were inversely correlated with the expression of two EGFR ligands, EREG and TGFA, and an EGFR dependent gene signature. Our results support the existence of a miR-203, EGFR, TKIs resistance regulatory network in prostate cancer progression. We propose that the loss of miR-203 is a molecular link in the progression of prostate cancer metastasis and TKIs resistance characterized by high EGFR ligands output and anti-apoptotic proteins activation.

## INTRODUCTION

Drug resistance and metastasis to the bone are the most clinically important features of prostate cancer, and understanding the signaling networks in these processes is central to developing improved outcomes for patients [1]. Epidermal growth factor receptor (EGFR) is a critical signaling molecule that controls several signaling pathways in prostate cancer [2, 3]. Binding of ligands such as amphiregulin (AREG), epiregulin (EREG), and transforming growth factor-α (TGFA) to the EGFR promotes the activation of downstream signaling pathways and induces cell survival, proliferation, invasion and metastasis [4]. Numerous studies have reported the activation of EGFR as a resistance mechanism to various anti-cancer therapies [1, 5-7]. Inhibition of EGFR using monoclonal antibodies (mAbs) to block receptor dimerization or small molecule tyrosine kinase inhibitors (TKIs) is a clinically relevant strategy for blocking EGFR signaling [8, 9]. However, there are various resistance mechanisms such as oncogenic bypass, which involves the activation of downstream molecular components of EGFR signaling [10, 11]. *KRAS*-mutant cancer is unresponsive to EGFR inhibitors because oncogenic KRAS is not dependent on upstream activation by EGFR [11, 12]. Activation of the Ras pathway is an essential step during tumorigenesis in humans, including metastatic prostate cancer [13, 14]. Importantly, Ras pathway activation up-regulates EGFR ligands, promoting an autocrine activation loop of EGFR signaling that is critical for tumor growth [15, 16].

In addition to Ras and EGFR, several microRNAs (miRs) have been shown to influence cancer development [17-19]. How miRs mediate EGFR signaling to modulate tumorigenesis is still ill-defined. miR-203 has been proposed as a tumor-suppressive microRNA in various types of cancer [20-23]. It has been shown in non-small cell lung cancer (NSCLC) that a receptor tyrosine kinase (RTK), *MET*, is a potential miR-203- targeted gene, and an inverse correlation between miR-203 and MET expression exists [21]. In addition,there is low miR-203 and high *SRC* expression in the majority of lung cancer tissues, and enforced expression of miR-203 clearly reduced the levels of SRC protein [21]. miR-203 is regulated by the protein kinase C/activator protein 1 (AP-1) pathway and activation of the EGFR pathway suppresses miR-203 expression in skin cancer [24]. Moreover, miR-203 was shown to repress endogenous *SNAI1/2*, forming a double negative miR203/Snail feedback loop in breast cancer [22]. In support of prostate cancer tumorigenesis, miR-203 has been found to be differentially down-regulated in bone metastatic prostate cancer cell lines and in clinical specimens [23]. However, it is unclear how miR-203 modulates EGFR signaling and how miR-203 regulates the expression of EGFR signaling-related genes in Ras-activated prostate cancer metastasis. The role of miR-203 in Ras-activated metastasis and EGFR inhibitor resistance in prostate cancer tumors remains largely unknown.

The activation of Ras pathway was shown to be significantly associated with TKIs resistance in NSCLC [25]. However, the intrinsic molecular mechanisms of resistance to these drugs in prostate cancer remain largely unknown. There is accumulating evidence showing that although resistance to apoptosis is a hallmark of cancer and can cause metastasis and resistance to drug treatment, cancer cells are typically targeting a small number of anti-apoptotic proteins for their survival [26, 27]. The most studied are the anti-apoptotic BCL-2 family members, inhibitor of apoptosis proteins, and the caspase inhibitors [28, 29]. Fewer reports have tackled the involvement of miRs in the regulation and the acquired sensitivity of TKIs-resistant prostate cancer. We show here that miR-203 expression suppresses bone metastasis and induces apoptosis in TKIs-resistant Ras-activated prostate cancer cells in which miR-203 is down-regulated by EGFR signaling. We determined that the 3'UTR of the mRNAs of EGFR ligands (*EREG* and *TGFA*) and anti-apoptotic proteins (*API5, BIRC2*, and *TRIAP1*) are direct targets of miR-203. Importantly, inhibition of miR-203 induced both cell invasion and resistance to TKIs treatment in prostate cancer cells, implying a dominant biological function for miR-203 in the EGFR network. These data suggest regulatory mechanisms whereby tumors with low miR-203 output, have an increase in EGFR ligands (*EREG* and *TGFA*) and anti-apoptotic proteins (*API5, BIRC2,* and *TRIAP1*) expression, resulting in prostate cancer bone metastasis and TKIs resistance.

## RESULTS

### Reduction of miR-203 expression is related to induced-EGFR signaling in Ras-activated prostate cancer cells

**Figure 1:**
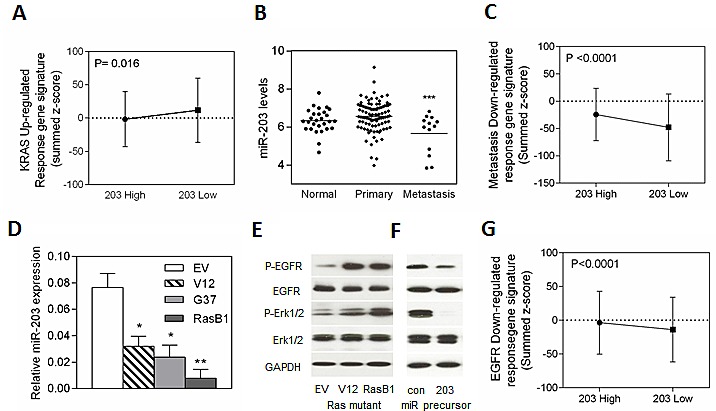
miR-203 expression is inversely correlated with a prostate cancer metastasis gene signature in the Taylor prostate dataset and EGFR signaling in an *in vitro* model (A) Mean summed z-scores for the KRAS signature in the human prostate carcinomas dataset segregated into high and low miR-203 expression where low miR-203 expressing patients have high expression of *KRAS* responsive genes signatures. (B) Mean miRNA expression of miR-203 in human normal (n=28), primary (n=98), and metastatic (n=13) prostate samples. Significance determined by one-way ANOVA. *: vs. primary. (C) Mean summed z-scores for the metastasis down regulated gene signature in the human prostate carcinoma set, showing that high miR-203 expressing patients have high expression of metastasis down-regulation responsive genes signatures. (D) qRT-PCR of miR-203 expression levels determined in DU145 cells with empty vector (EV), RasV12 (V12) or RasG37 (G37 and RasB1) mutant. miRNA expression was normalized to *SNORD48*. Data represent means ± SEM, n=3. *: vs. EV. (E and F) Representative Western Blot analysis of p-EGFR, EGFR, p-ERK1/2, and ERK1/2 in DU145 cells with empty vector (EV), RasV12 (V12) or RasG37 (RasB1) mutant transfection (E), or of RasB1 cells stably transfected with control and miR-203 microRNA precursor (F). (G) Mean summed z-scores for EGFR down-regulated signaling gene set in the Taylor clinical dataset, showing that high miR-203 expressing patients have high expression of the EGFR signaling down-regulation responsive genes signatures. *p<0.05. **p<0.01, ***p<0.001.

Although Ras mutations are relatively uncommon in prostate cancer [30], various lines of evidence suggest that RAS-dependent signaling contributes to the aggressiveness of advanced prostate cancer [31, 32]. Moreover, hyper-activation of the Ras signaling pathway have been proposed to promote prostate cancer progression and metastasis [33-35]. For example, loss of DAB2IP, a RasGTPase-activating protein (RasGAP), induced prostate cancer metastasis and its expression is inversely correlated with tumor grade and predicts prognosis [36]. In a non-metastatic human prostate cancer cell line (DU145), the effector pathways downstream of Ras have been assayed for their ability to promote metastasis [37]. To enrich for bone metastatic activity, DU145/RasG37 tumor cells were isolated from three independent bone metastases and expanded in culture [38]. Inoculation of the bone derived clones (DU145/RasB1), compared with the parental RasG37-transformed cells, demonstrated higher metastatic capacity as determined by a more rapid development of metastasis and formation of more and larger metastatic lesions [37]. miR-203 expression is reduced in clinical prostate cancer samples and in metastatic prostate cell lines [23]. Therefore, miR-203 could be a potential prognostic marker and therapeutic target in metastatic human prostate cancer. In order to identify whether miR-203 is linked to Ras signaling, we examined miR-203 expression levels and correlated mRNAs, in a publicly available data set of 99 primary tumors and 13 distant metastasis tissue specimens collected and analyzed at Memorial Sloan-Kettering Cancer Center [32]. The set was divided into two groups of ‘low’ and ‘high’ miR-203 expression level based on a measure of relative mRNA expression, z score [32]. An analysis of summed z-scores with a *KRAS* up-regulated gene set confirmed that miR-203 was expressed at low levels in tissues with altered KRAS signaling (Figure 1A). In addition, the actual mean intensity expression analysis in the clinical prostate database showed reduced miR-203 expression in metastatic tumor samples (Figure 1B). To test the relationship between miR-203 and prostate cancer metastasis, the miR-203 status assignments were validated by summed z-scores with a metastasis down-regulated response gene set. The results indicated that samples with high miR-203 levels showed an increase in metastasis down-regulated response gene set (Figure 1C). To further demonstrate that oncogenic KRAS represses miR-203 *in vitro*, we measured the expression of miR-203 in various DU145 cells that harbor different Ras mutations (V12, G37, and RasB1). As shown in Figure 1D, miR-203 expression was reduced in the DU145 cell line that harbors the RasG37 mutation and further reduced in the bone-derived clone (RasB1). The RasB1 cell line model has been shown to have a dramatic metastatic phenotype [37]. We observed that the cells with a Ras mutation had increased p-EGFR and p-ERK1/2 expression by Western Blot (WB) analysis (Figure 1E). To investigate the effect of miR-203 on the EGFR pathway, we forced the overexpression of miR-203 precursor in RasB1 cells. A reduction in p-EGFR and p-ERK1/2 expression levels was detected by WB analysis (Figure 1F). We also tested AR positive cell lines in response to miR-203 and detected a dramatic decrease in p-EGFR and p-ERK1/2 expression in LNCap cells (Figure S1A). Furthermore, an analysis of summed z-scores of EGFR pathway down-regulated response gene set showed that samples of increased miR-203 expression are highly associated with down-regulated EGFR pathway gene signatures expression (Figure 1G). This data suggests that miR-203 may target EGFR pathway related genes and may be down-regulated by EGFR signaling.

### Reduction of miR-203 regulates Ras-mutated prostate cancer cell growth and metastasis

**Figure 2:**
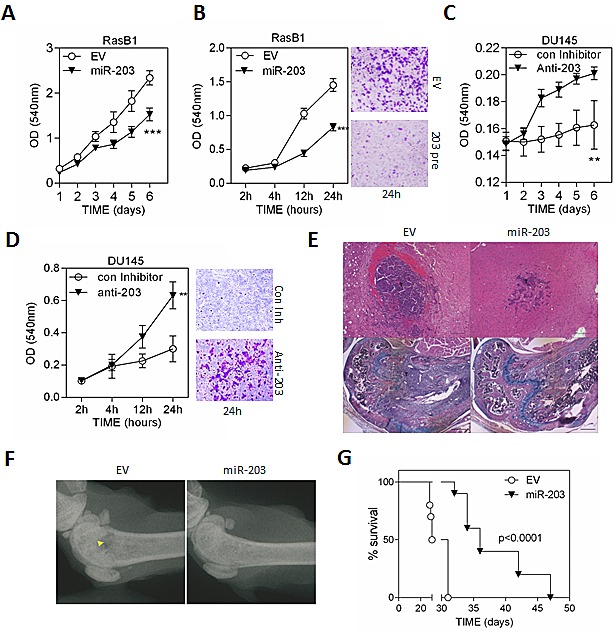
miR-203 inhibits cell metastasis of the RAS-activated prostate cancer cells, DU145-RasB1 (A) Representative data for *in vitro* growth curve of RasB1 cells expressing empty vector (EV) or miR-203 precursor for the indicated times and measured with ELISA reader at OD540nm. Data represent means ± SEM, n=5. *: vs. EV. (B) Cellular invasion of RasB1 cells infected with empty vector (EV) or miR-203 precursor lentivirus through Matrigel™-coated transwells for the indicated times, fixed and measured with ELISA reader at OD540nm. Data represent means ± SEM, n=5. *: vs. EV. (C and D) Cellular growth curve (C) and invasion (D) of DU145 cells transfected with 50nM of control or anti-203 inhibitor for the indicated times and measured with ELISA reader at OD540nm. Data represent means ± SEM, n=3. *: vs. control inhibitor. (E) Upper panels show brain metastasis of tumor bearing mice. Bottoms panels show bone metastasis in femur of tumor bearing mice. Tumor cells filled the bone marrow cavity in control (EV) bone with bone destruction. Both trabecular and cortical bones are destroyed. Scale bar: brain 100μm, bone 200μm. (F) Radiographic image of femurs from empty vector (EV) and miR-203 bearing mice. Yellow arrow indicates bone destruction. (G) Intra-cardiac injections of mice with RasB1 cells expressing empty vector or miR-203 precursor for the indicated times. Survival rate of tumor-bearing mice in each group (n=10). *p<0.05, **p<0.01, ***p<0.001.

In order to assess whether down-regulation of miR-203 is necessary for cellular transformation induced by oncogenic *KRAS* expression, we analyzed the functional effects of miR-203 on cell invasion and growth in Ras-mutated prostate cancer cells. The *in vitro* cell growth assay confirmed the significant effect of miR-203 overexpression on growth rate reduction in RasB1 cells (Figure 2A). In addition, we overexpressed miR-203 precursor in RasB1 cells and a reduction in cell invasion was obtained (Figure 2B). Importantly, inhibition of miR-203 in parental DU145 cells induced both cell growth and invasion *in vitro* (Figures 2C and 2D). To evaluate the effect of miR-203 on the metastatic efficiency of the well-established Ras-mutated bone metastatic prostate cancer cells *in vivo*, the miR-203 precursor was stably overexpressed in RasB1 cells. Using the intra-cardiac injection mouse model, RasB1 cells overexpressing miR-203 precursor showed a significant decrease in brain and bone metastasis (Figures 2E, 2F and S1B) and a significant increase in survival rate compared to the empty vector (Figures 1G and S1B). These data show that miR-203 suppresses a variety of metastasis properties as well as growth rate in advanced Ras-mutated prostate cancer cells.

### Activated EGFR signaling-induced autocrine *AREG, EREG*, and *TGFA* expression is associated with down-regulated miR-203

**Figure 3:**
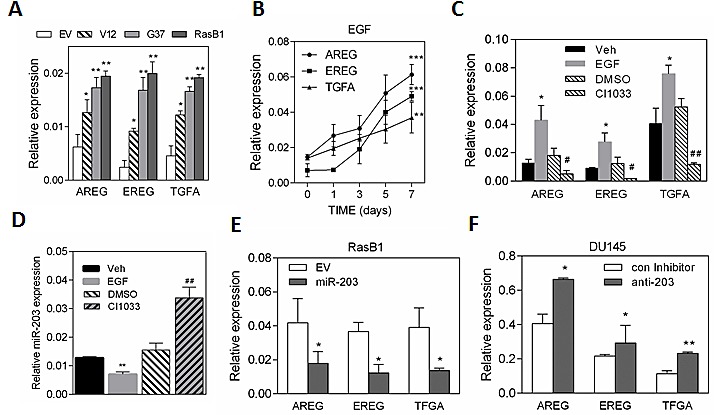
EGF has a positive feedback loop effect in up-regulating *AREG, EREG*, and T*GFA* expression in RasB1 cells (A) qRT-PCR of *AREG, EREG*, and *TGFA* levels determined in DU145 cells with empty vector (EV), RasV12 (V12) or RasG37 (G37 and RasB1) mutant. Relative mRNA expression was normalized to *GAPDH*. Data represent means ± SEM, n=3. *: vs. EV. (B) qRT-PCR analysis of *AREG, EREG,* and *TGFA* expression in RasB1 cells with EGF treatment for the indicated times. *: responsiveness to EGF. (C) qRT-PCR analysis of *AREG, EREG*, and *TGFA* levels in RasB1 cells with EGF or CI1033 treatment for 24 hours. *: vs. vehicle, #: vs. DMSO. (D) qRT-PCR analysis of miR-203 level in RasB1 cells after being treated with EGF or CI1033 for 24 hours. Relative mRNA expression was normalized to *SNORD48.* *: vs. vehicle, #: vs. DMSO. (E) qRT-PCR of *AREG, EREG*, and *TGFA* levels in RasB1 cells with empty vector (EV) or miR-203 precursor. Data represent means ± SEM, n=3. *: vs. EV. (F) qRT-PCR analysis of *AREG, EREG,* and *TGFA* expression in DU145 cells with an anti-miR inhibitor. Data represent means ± SEM, n=3. *: vs. control inhibitor. *p<0.05. **p<0.01, ***p<0.001.

Although Ras mutation in prostate cancer varies between populations, we hypothesized that persistent RAS activity might explain the induction of the EGFR signaling pathway in advanced prostate cancer cells. As expected, we found that RasB1 cells, harboring the RasG37 mutation, had increased mRNA expression levels of EGFR ligands, including *AREG, EREG,* and *TGFA* (Figure 3A). To determine whether EGF could induce the expression of *AREG, EREG,* and *TGFA*, kinetic analyses of EGF-treated RasB1 cells were performed. Increased expression of autocrine *AREG, EREG,* and *TGFA* are shown over time following EGF treatment of RasB1 cells (Figure 3B). In contrast, in the presence of EGFR inhibitor (CI1033), down-regulation of *AREG, EREG*, and *TGFA* expression was observed (Figure 3C). These data suggest that EGF has a positive feedback loop effect leading to the up-regulation of *AREG, EREG*, and *TGFA* expression. To examine the effect of EGF on the expression of miR-203, we treated RasB1 cells with EGF. The expression of miR-203 was markedly lower in the presence of EGF compared to untreated cells, however, it was up-regulated in CI1033-treated cells (Figure 3D). Furthermore, decreased mRNA levels of *AREG, EREG,* and *TGFA* were found in the presence of miR-203 precursor (Figure 3E), suggesting the presence of a miR-203 binding site on *AREG, EREG,* and *TGFA* 3'UTR. Importantly, inhibition of miR-203 in parental DU145 cells increased mRNA levels of *AREG, EREG,* and *TGFA* (Figure 3F). Markedly, our data demonstrate that the activated EGFR signaling-induced autocrine *AREG, EREG*, and *TGFA* expression in Ras-mutated prostate cancer cells is associated with a down-regulation in miR-203 expression level in an EGF-dependent manner.

### miR-203 directly binds to the 3'UTR of *AREG, EREG*, and *TGFA* and regulates the stability of *AREG, EREG*, and *TGFA* mRNA

**Figure 4:**
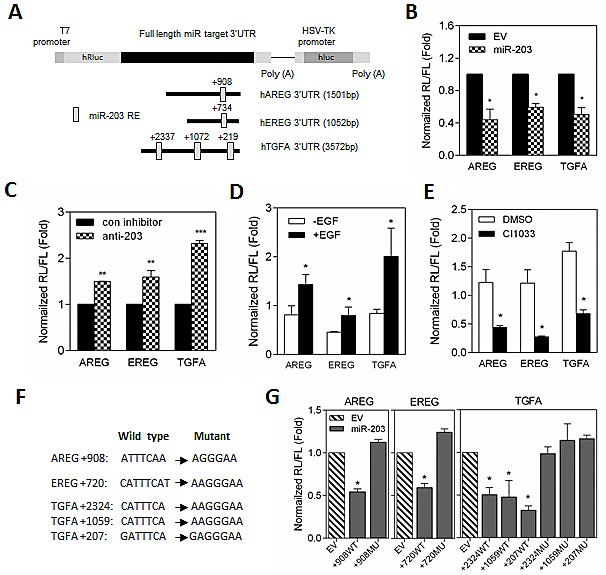
The stability of A*REG, EREG*, and *TGFA* mRNA is directly regulated by miR-203 (A) Schematic of predicted miR-203 binding sites in full-length 3'UTR reporter constructs of human *AREG, EREG,* and *TGFA*. (B) The normalized reporter activity of miRNA target reporters containing the full length human *AREG, EREG*, and *TGFA* 3'UTRs in RasB1 cells with transient expression of empty vector (EV) or miR-203 precursor. Renilla/luciferase activities were measured 48 hours after transfection. Data represent means ± SEM of separate transfections, n=3. *: vs. EV. (C) The normalized reporter activity of miRNA target reporters containing the full length human *AREG, EREG,* and *TGFA* 3'UTRs in DU145 cells with transient expression of control or anti-miR-203 inhibitor. *: vs. control inhibitor. (D and E) The normalized reporter activity of full length *AREG, EREG,* and *TGFA* 3'UTRs in RasB1 cells with EGF (D) or CI1033 (E) treatment. Data represent means ± SEM, n=3. *: vs.-EGF or DMSO. (F) Schematic of the predicted, conserved miR-203 binding sites and the introduced binding site mutants in the *AREG, EREG,* and *TGFA* 3'UTRs reporters. (G) The normalized reporter activity of *AREG, EREG,* and *TGFA* 3'UTR containing wild type or mutated miRNA target reporters in RasB1 cells with transient expression of empty vector or miR-203 precursor. Renilla/luciferase activities were measured 48 hours after transfection. Data represent means ± SEM of separate transfections, n=3. *: vs. EV. *p<0.05. **p<0.01, ***p<0.001.

Given the critical role of Ras mutation-induced EGFR signaling activation in prostate cancer progression, we hypothesized that EGF-induced autocrine *AREG, EREG,* and *TGFA* expression might be directly mediated by miR-203 in RasB1 cells. To further investigate the presence of miR-203 binding sites, the homologous binding sites of miR-203 in full length *AREG, EREG*, and *TGFA* 3'UTR were analyzed (Figure 4A), and decreased luciferase activities were detected upon co-transfection with miR-203 precursor by reporter assay, respectively (Figure 4B). In addition, inhibition of miR-203 in parental DU145 cells induced 3'UTR reporter activities of *AREG, EREG,* and *TGFA* (Figure 4C). Furthermore, EGF-treated RasB1 cells showed a significant increase in *AREG, EREG,* and *TGFA* 3'UTR reporter activity (Figure 4D) and a decreased reporter activity upon CI1033 treatment (Figure 4E). These data suggest that EGF-reduced miR-203 expression may directly mediate *AREG, EREG*, and *TGFA* expression in RasB1 cells. To further determine the relative contribution of miR-203-dependent regulation, individual response elements and mutations of miR-203 target sites reporter constructs were prepared (Figure 4F). Reporter assays demonstrated a specific repressive role for miR-203 on each binding site (Figure 4G). These data suggest that miR-203 directly binds to the 3'UTR of *AREG, EREG*, and *TGFA* and regulates the stability of mRNA.

### Increased *AREG, EREG*, and *TGFA* expression is related to reduced miR-203 expression and activation of RAS signaling in metastatic prostate cancer patients

**Figure 5:**
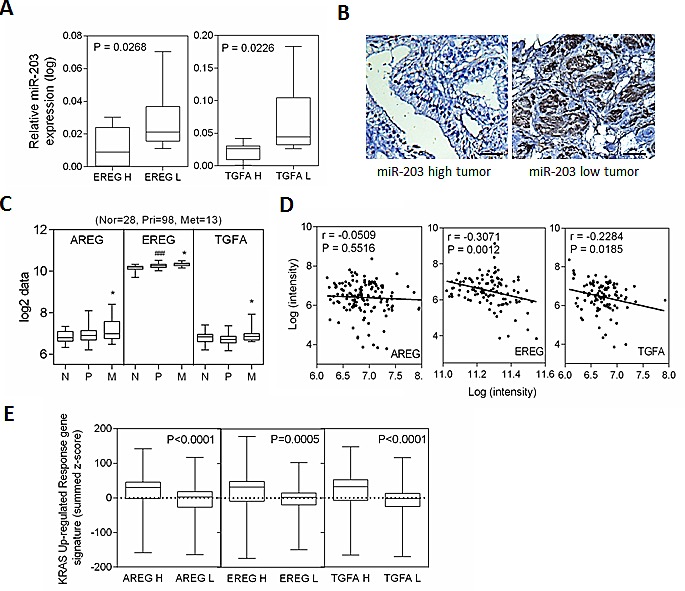
miR-203 induces decreased levels of *AREG, EREG*, and *TGFA* expression in the progression of prostate cancer (A) qRT-PCR of miR-203 levels determined in 25 individuals with prostate cancer. Real-time PCR was used to classify tumors into two groups, low and high of *EREG* and *TGFA*. Significance determined by the Student's t test. (B) Representative immunohistochemical staining with antibodies specific for EREG are shown for 25 individual tissue sections from miR-203 high and low prostate cancers. Scale bars represent 100μm. (C) Mean mRNA expression of *AREG* and *EREG* in human normal (n=28), primary (n=98), and metastatic (n=13) prostate samples from the Taylor dataset. Significance determined by one-way ANOVA. *: vs. primary, #: vs. normal. (D) Pearson anti-correlation coefficient of mean miR-203 to mean *AREG* or *EREG* mRNA expression in primary and metastasis prostate samples (n=111). Significance determined by Gaussian population (Pearson) and two-tailed test. (E) Mean summed z-scores for the EGFR ligands (*AREG, EGEG*, and *TGFA*) signature in human prostate carcinomas dataset of *KRAS* responsive genes set, showing that high *AREG, EGEG*, and *TGFA* expressing patients have high expression of *KRAS* up-regulated responsive genes signatures. *p<0.05. **p<0.01, ***p<0.001.

To further study the inverse correlation between miR-203 and its targets in human prostate tissue, we analyzed 25 independent prostate tumors collected form Wan Fang Hospital, Taipei Medical University, Taiwan, which we divided into two groups of ‘low’ and ‘high’ *EREG* and *TGFA* expression level, based on qRT-PCR analyses. An analysis of variance confirmed that the miR-203 was differentially expressed between the low and high groups, where tissues with high levels of *EREG* and *TGFA* expression had lower miR-203 expression (Figure 5A). In addition, immunohistochemistry analysis of tumors with low miR-203 expression showed EREG overexpression in tumors that had distant metastases compared to tumors with high miR-203 expression (Figure 5B). We explored the relevance of this finding to study the activation of EGFR ligands in a public human prostate cancer dataset [32]. The actual mean intensity expression analysis in the clinical prostate database showed that *AREG, EREG*, and *TGFA* levels increased in metastatic tumor samples (Figure 5C). To examine the inverse correlation between miR-203 and EGFR ligands expression in prostate cancer progression, we analyzed the mean expression of miR-203 and EGFR ligands in primary and metastatic prostate cancer samples. Decreased mean expression of *EREG* and *TGFA* were significantly inversely correlated to miR-203 but not *AREG* by Pearson coefficient correction analysis (Figure 5D). Moreover, we observed a high expression of *AREG, EREG*, and *TGFA* in tumors that had higher *KRAS* oncogenic response gene signatures in the human prostate cancer dataset [32] (Figure 5E). These results are consistent with our observation linking miR-203 inactivation with a significantly increased EGFR ligand expression required in oncogenic KRAS activated prostate cancer.

### miR-203 overexpression contributes to the induction of apoptosis in TKI-resistant RAS-activated prostate cancer cells

**Figure 6:**
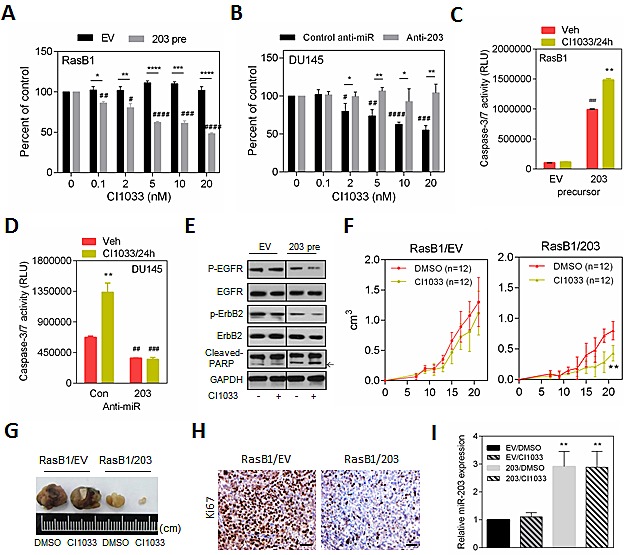
miR-203 induces apoptosis in the TKI-resistant RAS-activated prostate cancer cells (A) RasB1 cells with empty vector (EV) and with miR-203 precursor were treated with increasing concentrations of CI1033. Cell viability, relative to untreated controls, was measured at 24 hour. Each data point represents the mean± s.d. of six wells. #: vs. 0 nM, *: vs. EV. (B) DU145 cells with control anti-miR and with anti-miR-203 inhibitor were treated with increasing concentrations of CI1033. Cell viability, relative to untreated controls, was measured at 24 hour. Each data point represents the mean± s.d. of six wells. #: vs. 0 nM, *:vs. control anti-miR. (C) RasB1 cells were transfected with empty vector (EV) and with miR-203 precursor, and 24 hours after being treated with CI1033, apoptosis was assessed by measuring caspase-3/7 activity with relative luciferase unit (RLU). Data represent means ± SEM, n=3. #: vs. EV, *: vs. vehicle. (D) DU145 cells were transfected with control anti-miR and with anti-miR-203 inhibitor, and 24 hours after being treated with CI1033, apoptosis was assessed by measuring caspase-3/7 activity with relative luciferase unit (RLU). #: vs. control anti-miR, *: vs. vehicle. (E) Representative Western Blot analysis of P-EGFR, EGFR, P-ErbB2, ErbB2, and cleaved-PARP in RasB1 cells stably transfected with empty vector (EV) or miR-203 precursor, and 24 hours treatment with CI1033 or DMSO. (F and G) Growth curve of engrafted subcutaneous tumors (F) and comparison of engrafted tumors (G) in nude mice injected with RasB1 cells stably infected with precursor of miR-203 or an empty vector as a control. The images show average-sized tumors selected from ten tumors per category after treatment with 20mg/kg CI1033 or DMSO as control. (H) Representative immunohistochemical staining with antibody specific for Ki67 are shown for tissue sections from RasB1 cells expressing empty vector or miR-203 precursor. Scale bars represent 100μm. (I) Confirmed up-regulation of the miR-203 in the xenograft tumors by qRT-PCR. qRT-PCR analysis of miR-203 measured in RNA isolated from subcutaneous tumors formed by genetically-altered RasB1 cells as indicated in Figure 6G. Data represent means ± SEM, n=3. *: vs. EV/DMSO. *p<0.05. **p<0.01, ***p<0.001.

It has been suggested and shown that the activation of the Ras pathway is significantly associated with TKIs-resistance in NSCLC [25]. Therefore, we sought to study the effect of miR-203 expression and its contribution to apoptosis in TKIs-resistant Ras-activated prostate cancer cells. EGFR inhibitor (CI1033 or AG1478) treatment of RasB1 cells, transfected with an empty vector,demonstrated resistance to cell death relative to untreated controls. However, the level of cell death of miR-203 precursor overexpressing cells was significantly increased in a dose-dependent manner when compared to RasB1 cells transfected with an empty vector (Figures 6A and S2A). These results suggest that miR-203 overexpression induces sensitivity to TKIs treatment in previously TKIs-resistant RasB1 cells. To determine the relative contribution of miR-203 in regulating apoptosis upon EGFR inhibitor treatment, parental DU145 cells were transfected with anti-miR-203. Cell viability assays demonstrated a dose-dependent effect on cell death for cells transfected with the control anti-miR, however, a resistance effect to CI1033 or AG1478 treatment was detected in cells transfected with anti-miR-203 (Figures 6B and S2B). These data suggest that the effect of EGFR inhibitors on cell viability and cell death is miR-203-dependent. Thus, miR-203 can directly attenuate the sensitivity of cells to EGFR inhibitors. Furthermore, we observed TKI-induced caspase-3/7 activities in TKIs-resistant RasB1 cells overexpressing miR-203, but not in cells transfected with the empty vector plasmid (Figure 6C). In contrast, in the parental DU145 cells, decreased caspase-3/7 activity was shown in cells transfected with anti-miR-203 in the presence or absence of CI1033, when compared to control anti-miR (Figure 6D). Importantly, TKI-induced caspase-3/7 activity was not detected in DU145 cells transfected with anti-miR-203 (Figure 6D). Moreover, following treatment with CI1033, RasB1 cells harboring the miR-203 precursor had increased cleaved-PARP expression (Figure 6E). Confirming the hypothesis of the involvement of other EGFR-related family members in the resistance process in our cell system, the results showed that other EGFR-related family members, such as ErbB2, are involved in this resistance process in RasB1 cells (Figure 6E). Furthermore, inhibition of endogenous miR-203 in DU145, using anti-miR-203, affected EGFR signaling pathways and reduced the expression of cleaved-PARP in the presence of CI1033, suggesting that inhibition of endogenous miR-203 in DU145 activates EGFR signaling and induces drug resistance (Figure S2C). To further confirm the relevance of miR-203 in TKIs-induced apoptosis *in vivo*, the overexpression of miR-203 in RasB1 cells resulted in a marked inhibition of tumor growth and increased sensitivity to TKI-induced apoptosis in nude mice after 3 weeks of CI1033 treatment (Figures 6F and 6G). In addition to that, a reduction in Ki67-positive cells in xenograft tumors overexpressing miR-203 was observed (Figure 6H). The expression of miR-203 in the tumors was confirmed by qRT-PCR (Figure 6I). These results suggest that the effects of miR-203 on TKIs sensitivity are due to directly regulating the anti-apoptotic signaling-related genes mediated by miR-203.

### Activated EGFR/RAS signaling induces an anti-apoptotic pathway that is regulated by miR-203

**Figure 7:**
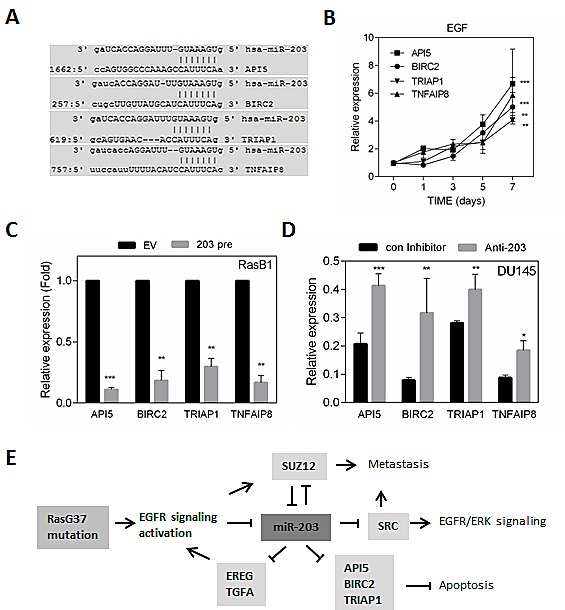
miR-203 regulates anti-apoptotic proteins and oncogenic molecule in KRAS activated prostate cancer cells (A) Schematic of the predicted, conserved miR-203 binding site in the 3'UTR of *API5, BIRC2, TRIAP1,* and *TNFAIP8*. (B) qRT-PCR analysis of *API5, BIRC2, TRIAP1,* and *TNFAIP8* expression in RasB1 cells with EGF treatment at indicated times is shown. (C) qRT-PCR analysis of *API5, BIRC2, TRIAP1,* and *TNFAIP8* in RasB1 cells with empty vector (EV) or miR-203 precursor. Relative mRNA expression was normalized to *GAPDH*. Data represent means ± SEM, n=3. *: vs. EV. (D) qRT-PCR analysis of *API5, BIRC2, TRIAP1*, and *TNFAIP8* expression in DU145 cells with control or anti-miR-203 inhibitor. Data represent means ± SEM, n=3. *: vs. control inhibitor. *p<0.05, **p<0.01, ***p<0.001. (E) A proposed model describing the interactions of miR-203 with EGFR ligands and anti-apoptotic proteins leading to inhibition of apoptosis, TKIs resistance, and metastasis in RAS-activated prostate cancer.

To validate miR-203 as a regulator of TKI resistance via an apoptotic pathway, we assayed a number of predicted miR-203 targets that are associated with apoptosis signaling (Figure 7A). We examined whether EGF could induce the expression of the expected miR-203 targets. Increased expression of anti-apoptotic proteins, *API5, BIRC2,* and *TRIAP1* are shown over time following EGF treatment of RasB1 cells (Figure 7B). Furthermore, *API5, BIRC2,* and *TRIAP1* were markedly reduced in the presence of miR-203 precursor as assayed in RasB1 cells (Figure 7C). In contrast, in the parental DU145 cells, in the presence of anti-miR-203, up-regulation of *API5, BIRC2,* and *TRIAP1* expression was observed (Figure 7D). These data suggest that miR-203 has a critical effect in down-regulating mRNA expression of the anti-apoptotic proteins, *API5, BIRC2*, and *TRIAP1*. In addition to anti-apoptotic proteins, an NF-κB-inducible, oncogenic molecule, *TNFAIP8* was significantly affected by miR-203 expression in cells with forced expression of each of the precursor or inhibitor (Figures 7C and 7D). Taken together, it appears that miR-203 regulates anti-apoptotic proteins as well as an oncogenic molecule and acts independently as a tumor suppressor by regulating additional targets in KRAS-activated prostate cancer cells. Our results show that the induction of TKIs resistance functions through EGFR signaling activation by the down-regulation of miR-203-mediated expression of the EGFR ligands *EREG* and *TGFA,and the* anti-apoptotic proteins *API5, BIRC2,* and *TRIAP1*, as schematically depicted in Figure 7E.

## DISCUSSION

In this study, we investigated the molecular mechanism of prostate cancer metastasis and identified novel roles of. genes that are regulated by miR-203 in Ras-activated prostate cancer. We identified the regulatory mechanisms of miR-203 interaction with EGFR signaling, and we defined the roles of the miR-203 regulatory pathway in Ras-activated prostate cancer metastasis and tyrosine kinase inhibitors (TKIs) resistance processes. A strong correlation between EGFR activation and several signaling pathways, such as RAS, AKT, and MAPK has been reported in prostate cancer [14]. Here we described a RAS mutation (RasG37 mutant) model of progressive prostate cancer that is associated with EGFR signaling activation and miR-203 inhibition, leading to the up-regulation of EGFR ligands. Since the RasG37 mutation does not strongly activate the RAF-ERK pathway, miR-203 suppression of ERK phosphorylation in the cell line might be through critical targets that reside downstream of EGFR. Our results are consistent with others that show that *SRC* is a potential miR-203 target gene [21], where we showed an inverse correlation between miR-203 and *SRC* expression in a RAS mutation prostate cancer model (Figures S3). Moreover, SRC is downstream of EGFR and upstream of signaling molecules such as AKT and ERK. The SRC mechanism of triggering EGFR signaling (phosphorylated EGFR and ERK1/2) is by miR-203 inhibition, which provides the direct mechanistic linkage between the miR-203-regulated *SRC* and EGFR signaling. We investigated the regulatory mechanisms of miR-203 signaling pathways that are important for RasG37-driven prostate cancer metastasis and clarified the physiological relevance of this line of investigation. The EGFR pathway is the dominant regulator of prostate cancer progression, and so we analyzed the regulatory interactions of EGFR ligands with miR-203 and its respective functional roles in TKIs resistance and metastasis. We showed an amplifying regulatory loop involving the direct interaction of miR-203 with the 3'UTR of EGFR ligands, *AREG, EREG*, and *TGFA*. In addition, we determined the mechanisms by which miR-203 overexpression contributes to TKIs-resistant Ras-activated prostate cancer cell apoptosis by targeting the 3'UTR of anti-apoptotic proteins, *API5, BIRC2*, and *TRIAP1*. Using a RAS mutation model, our study provides a novel functional link between miR-203 loss and increased EGFR ligands and shows how miR-203 regulates EGFR signaling response genes in prostate cancer metastasis and TKIs resistance.

The polycomb repressive complexes PRC1 and PRC2 are well known for remodeling chromatin structure by epigenetic silencing of genes during early development [39]. The expression of Suz12, member of the PRC2, is up-regulated in various cancers such as melanoma, lymphoma, breast, and prostate cancer [40]. miR-203 was shown to repress endogenous *SNAI1/2*, forming a double negative miR203/Snail feedback loop in breast cancer [22]. Noticeably, we established a relationship between the effects of Snail associated with Suz12 regulated by EGFR signaling in Ras-activated prostate cancer (Figures S4), and we have deciphered a novel role of miR-203 in regulating *SUZ12* expression in prostate cancer via direct interaction with *SUZ12* 3'UTR (Figures S5). Consistent with a previous report, although Snail has been shown to interact with Suz12 by binding to the CDH1 promoter [41], our data showed that the associated Snail/Suz12 directly bind to E-boxes in the primary *has-mir-203a* stem-loop promoter and function to inhibit miR-203 transcription in an EGF-dependent manner (Figures S6). Bmi1, member of the PRC1, has also been found to be a candidate target gene of miR-203, where ectopic expression of miR-203 in pancreatic and colorectal cancer cells induced apoptosis and repressed cell growth [42]. Likewise, overexpression of miR-203 inhibits breast and prostate cancer cells invasion through targeting 3'UTR of the mRNA of PRC1 [43]. Our data confirm the roles of miR-203 in regulating the expression of PRCs and provide evidence showing that activated EGFR signaling induces Snail/PRCs expression and down-regulates *has-mir-203a* stem-loop transactivation thereby silencing miR-203 expression, which is consistent with a critical role of miR-203 in cancer cells.

There have been previous hints of a connection between miR-203 and apoptosis. For instance, the anti-apoptotic proteins *SOCS3, BCL2*, and *BIRC5* are known to be implicated in a number of cancers and to contain putative miR-203 binding sites within their 3'UTRs [23, 44]. We have shown that miR-203 influenced the mRNA stability of candidate miR-203 targets that are either anti-apoptotic proteins (e.g. *API5, BIRC2,* and *TRIAP1*) or the novel oncogenic molecule: *TNFAIP8*. *TNFAIP8* is an NF-κB-inducible molecule that has been shown to be overexpressed in high-grade prostatic adenocarcinomas [45]. Our study demonstrated that *TNFAIP8* is a unique miR-203 target in RAS-mutated prostate cancer cells. The role of *API5*, a novel candidate target of miR-203, is potentially interesting since *API5* encodes an apoptosis inhibitory protein that suppresses the transcription factor E2F1 [46]. E2F1 induces apoptosis and acts as a tumor suppressor in retinoblastoma [47]. Therefore, the ability to down-regulate *API5* can contribute to the apoptotic activity of miR-203 and its effects on tumor cells. These observations suggest that the latter category may be synergistically affected by the regulatory loop of miR-203 depletion and anti-apoptotic proteins overexpression.

The overexpression of EGFR has been shown in a majority of cases of prostate cancer. Schlomm et al. reported that EGFR expression was found in 18% of prostate cancer and was associated with high grade, advanced stages, and high risk for prostate-specific antigen recurrence [48]. In addition, the expression of EGFR is correlated with a risk of recurrence and progression to hormone resistance. Furthermore, it has been shown that 100% of metastatic, castration-resistant prostate cancers (CRPC) express EGFR, suggesting that EGFR signaling plays an important role in the progression of prostate cancer [49, 50]. Critical EGFR-dependent signaling proceeds through its proximal downstream effectors, including notably the Ras group of paralogs (K-, H-, and N-Ras) [51]. Activation of the Ras pathway is commonly observed to be associated with prostate cancer progression [31, 32]. For the KRAS gene, the frequency of mutation in relation to prostate cancer has been reported across a range of geographical regions, races and patient cohorts. The mutation frequency of KRAS ranged from 3 to 16% in the Asian population [52-54], but was below 4% in western patients with prostate cancer [55, 56]. Our study describes how miR-203 functions in prostate cancer development and how it could be used as a diagnostic and prognostic marker. Most importantly, our findings will contribute to a better understanding to further justify the clinical use of EGFR inhibitors in patients with KRAS mutation in prostate cancer.

In conclusion, our results provide evidence showing that miR-203 functions as a tumor suppressor in RAS-dependent prostate cancer metastasis, and explore the regulatory mechanisms of how miR-203 is regulated through the TKIs resistance. The results from the analysis of clinical specimens suggest that decreasing miR-203 and increasing of EGFR ligands, (*EREG* and *TGFA) expression* are correlated with prostate cancer progression. Moreover, our results also identified miR-203 target sites in the 3'UTRs of *AREG, EREG*, and *TGFA*. Decreased mRNA levels of these EGFR ligands (*AREG, EREG*, and *TGFA*) and anti-apoptotic proteins (*API5, BIRC2,* and *TRIAP1*) were found in the presence of miR-203 precursor. Most importantly, our study contributes to a better understanding of prostate cancer metastasis processes at the molecular level. This may help clinical oncologists in planning an alternative therapeutic strategy to deal with the thorny problems of tumor metastasis. Our study demonstrated that the modulation of a specific miRNA might provide a therapeutic approach for the treatment of RAS-activated prostate cancer patients.

## MATERIALS AND METHODS

### Reagents and Constructs

EGF was from R&D (R&D Systems, MN), and EGFR inhibitor (CI1033 and AG1478) was from Selleck (Selleck, TX). The BD Matrigel™ was purchased from BD Biosciences (BD Biosciences, CA) for invasion assay and tumor cell injections. Precursor for miRNAs (empty vector and miR-203 precursor) and anti-miR inhibitors (control and anti-miR-203) were from GeneCopoeia (GeneCopoeia, MD). RFP reporter vectors were constructed using the Clone-it Enzyme free Lenti-vectors Kit (System Biosciences, CA). Human *AREG, EREG*, and *TGFA* full length 3'UTR reporters were constructed using the psiHECKTM-2 vector (Promega, WI). The microRNA binding site mutations were made using the Site-Directed Mutagenesis System kit (Invitrogen, CA). All primers used for these constructs are listed in Supplemental Tables (Table S1). All constructs were verified by DNA sequence analysis.

### Cell Culture

The DU145/RasG37 cell line was modified from the human prostate cancer cell line DU145 by stably expressing RasG37 mutation construct. RasB1 cell line was isolated from DU145/RasG37 orthotropic injection bone metastatic site [37]. DU145 and DU145 modified cell lines were cultured in RPMI 1640 media supplemented with 10% FCS. Transient transfections were carried out using Lipofectamine RNAiMAX (Invitrogen, CA). Dosage of EGF and EGFR inhibitor was EGF (10μM), and CI1033 (10nM) in serum free condition.

### Real-time RT-PCR

Total RNA was isolated using mirVana PARIS RNA isolation system (Ambion, TX). Reverse transcription of cDNA and PCR were performed as described in Supplemental Materials and Methods. All primers used for PCR are listed in Supplemental Tables (Table S2).

### Western Blot Analysis

Cells were lysed with RIPA buffer containing complete protease inhibitors (Roche, CA) plus the phosphatase inhibitors (Roche, CA), 25mM β-glycerophosphate, 10mM sodium fluoride and 1 mM Sodium Vanadate were performed as described [57, 58]. Primary antibodies were incubated overnight at 4°C using the dilutions listed in Supplemental Tables (Table S3).

### MicroRNA Luciferase Assay

MicroRNA luciferase assays were performed as described in Supplemental Materials and Methods. The microRNA binding sites on human *AREG, EREG,* and *TGFA* 3'UTR were determined using the Computational Biology Center, Memorial Sloan Kettering Cancer Center website (microRNA.org) and Bioinformatics and Research Computing, Whitehead Institute for Biomedical Research (TargetScan.org).

### *In vitro* Cell death and Proliferation Assay

For detection of caspase-3/7 activity, cells were cultured in 96-well plates, in triplicate, treated with 10nM CI1033 or 10μM AG1478 and analyzed using a Caspase-Glo 3/7 Assay kit (Promega, WI) according to the manufacturer's instructions. Continuous variables are expressed as means ± s.d. Cell viability was examined with 3-(4,5-dimethylthiazol-2-yl)-2,5-dipheniltetrazolium bromide (MTS)-Cell Titer 96 AQueous One Solution Cell Proliferation Assay (Promega, WI) according to the manufacturer's protocol. In vitro growth curves were performed as described previously [57, 58] using a density of 2X10^3^ cells per well.

### Invasion Assay

Cells that invaded the Matrigel™ (Falcon, NJ)-coated transwells in response to 10% FCS after 2, 4, 12, and 24 hours were fixed and counted as described [57, 58].

### Immunohistochemistry (IHC) staining

The clinical samples used 25 independent primary prostate tumors were collected from Wan Fang Hospital, Taipei Medical University, Taiwan. Immunohistochemistry (IHC) was performed using the EREG antibodies from R&D (R&D Systems, MN) at 1:60 dilution and staining was as described in Supplemental Materials and Methods.

### Animal Studies

Animal work was performed in accordance with a protocol approved by the TMU Animal Care and Use Committee. To analyze tumorigenesis, five-week old male nude mice (NLAC, Taipei) were injected subcutaneously with 1X10^6^ tumor cells in 50% Matrigel™ (Falcon, NJ). Intracardiac inoculation and bioluminescent imaging (BLI) were as described [37]. For survival studies, mice were euthanized when one of the following situations applied: 10% loss of body weight, paralysis, or head tilting. All animal studies were repeated three times.

## SUPPLEMENTAL MATERIALS AND METHODS



## References

[1] Holohan C, Van Schaeybroeck S, Longley DB, Johnston PG (2013). Cancer drug resistance: An evolving paradigm. Nat Rev Cancer.

[2] Di Lorenzo G, Tortora G, D'Armiento FP, De Rosa G, Staibano S, Autorino R, D'Armiento M, De Laurentiis M, De Placido S, Catalano G, Bianco AR, Ciardiello F (2002). Expression of epidermal growth factor receptor correlates with disease relapse and progression to androgen-independence in human prostate cancer. Clin Cancer Res.

[3] Traish AM, Morgentaler A (2009). Epidermal growth factor receptor expression escapes androgen regulation in prostate cancer: A potential molecular switch for tumour growth. Br J Cancer.

[4] Ferguson KM, Berger MB, Mendrola JM, Cho HS, Leahy DJ, Lemmon MA (2003). Egf activates its receptor by removing interactions that autoinhibit ectodomain dimerization. Mol Cell.

[5] Lynch TJ, Bell DW, Sordella R, Gurubhagavatula S, Okimoto RA, Brannigan BW, Harris PL, Haserlat SM, Supko JG, Haluska FG, Louis DN, Christiani DC, Settleman J, Haber DA (2004). Activating mutations in the epidermal growth factor receptor underlying responsiveness of non-small-cell lung cancer to gefitinib. N Engl J Med.

[6] Paez JG, Janne PA, Lee JC, Tracy S, Greulich H, Gabriel S, Herman P, Kaye FJ, Lindeman N, Boggon TJ, Naoki K, Sasaki H, Fujii Y, Eck MJ, Sellers WR, Johnson BE, Meyerson M (2004). Egfr mutations in lung cancer: Correlation with clinical response to gefitinib therapy. Science.

[7] Nathanson DA, Gini B, Mottahedeh J, Visnyei K, Koga T, Gomez G, Eskin A, Hwang K, Wang J, Masui K, Paucar A, Yang H, Ohashi M, Zhu S, Wykosky J, Reed R, Nelson SF, Cloughesy TF, James CD, Rao PN, Kornblum HI, Heath JR, Cavenee WK, Furnari FB, Mischel PS (2014). Targeted therapy resistance mediated by dynamic regulation of extrachromosomal mutant egfr DNA. Science.

[8] Matar P, Rojo F, Cassia R, Moreno-Bueno G, Di Cosimo S, Tabernero J, Guzman M, Rodriguez S, Arribas J, Palacios J, Baselga J (2004). Combined epidermal growth factor receptor targeting with the tyrosine kinase inhibitor gefitinib (zd1839) and the monoclonal antibody cetuximab (imc-c225): Superiority over single-agent receptor targeting. Clin Cancer Res.

[9] Gelsomino F, Agustoni F, Niger M, Valota M, Haspinger ER (2013). Epidermal growth factor receptor tyrosine kinase inhibitor treatment in patients with egfr wild-type non-small-cell lung cancer: The never-ending story. J Clin Oncol.

[10] Sergina NV, Rausch M, Wang D, Blair J, Hann B, Shokat KM, Moasser MM (2007). Escape from her-family tyrosine kinase inhibitor therapy by the kinase-inactive her3. Nature.

[11] Engelman JA, Zejnullahu K, Mitsudomi T, Song Y, Hyland C, Park JO, Lindeman N, Gale CM, Zhao X, Christensen J, Kosaka T, Holmes AJ, Rogers AM, Cappuzzo F, Mok T, Lee C, Johnson BE, Cantley LC, Janne PA (2007). Met amplification leads to gefitinib resistance in lung cancer by activating erbb3 signaling. Science.

[12] Nazarian R, Shi H, Wang Q, Kong X, Koya RC, Lee H, Chen Z, Lee MK, Attar N, Sazegar H, Chodon T, Nelson SF, McArthur G, Sosman JA, Ribas A, Lo RS (2010). Melanomas acquire resistance to b-raf(v600e) inhibition by rtk or n-ras upregulation. Nature.

[13] Davies H, Bignell GR, Cox C, Stephens P, Edkins S, Clegg S, Teague J, Woffendin H, Garnett MJ, Bottomley W, Davis N, Dicks E, Ewing R, Floyd Y, Gray K, Hall S, Hawes R, Hughes J, Kosmidou V, Menzies A, Mould C, Parker A, Stevens C, Watt S, Hooper S, Wilson R, Jayatilake H, Gusterson BA, Cooper C, Shipley J, Hargrave D, Pritchard-Jones K, Maitland N, Chenevix-Trench G, Riggins GJ, Bigner DD, Palmieri G, Cossu A, Flanagan A, Nicholson A, Ho JW, Leung SY, Yuen ST, Weber BL, Seigler HF, Darrow TL, Paterson H, Marais R, Marshall CJ, Wooster R, Stratton MR, Futreal PA (2002). Mutations of the braf gene in human cancer. Nature.

[14] Gan Y, Shi C, Inge L, Hibner M, Balducci J, Huang Y (2010). Differential roles of erk and akt pathways in regulation of egfr-mediated signaling and motility in prostate cancer cells. Oncogene.

[15] Schulze A, Nicke B, Warne PH, Tomlinson S, Downward J (2004). The transcriptional response to raf activation is almost completely dependent on mitogen-activated protein kinase kinase activity and shows a major autocrine component. Mol Biol Cell.

[16] McCarthy SA, Samuels ML, Pritchard CA, Abraham JA, McMahon M (1995). Rapid induction of heparin-binding epidermal growth factor/diphtheria toxin receptor expression by raf and ras oncogenes. Genes Dev.

[17] Bartel DP (2009). Micrornas: Target recognition and regulatory functions. Cell.

[18] Nicoloso MS, Spizzo R, Shimizu M, Rossi S, Calin GA (2009). Micrornas--the micro steering wheel of tumour metastases. Nat Rev Cancer.

[19] Lujambio A, Lowe SW (2012). The microcosmos of cancer. Nature.

[20] Lena AM, Shalom-Feuerstein R, Rivetti di Val Cervo P, Aberdam D, Knight RA, Melino G, Candi E (2008). Mir-203 represses ‘stemness’ by repressing deltanp63. Cell Death Differ.

[21] Garofalo M, Romano G, Di Leva G, Nuovo G, Jeon YJ, Ngankeu A, Sun J, Lovat F, Alder H, Condorelli G, Engelman JA, Ono M, Rho JK, Cascione L, Volinia S, Nephew KP, Croce CM (2011). Egfr and met receptor tyrosine kinase-altered microrna expression induces tumorigenesis and gefitinib resistance in lung cancers. Nat Med.

[22] Moes M, Le BéchecLe A, Crespo I, Laurini C, Halavatyi A, Vetter G, del Sol A, Friederich E (2012). A novel network integrating a mirna-203/snai1 feedback loop which regulates epithelial to mesenchymal transition. PLoS One.

[23] Saini S, Majid S, Yamamura S, Tabatabai L, Suh SO, Shahryari V, Chen Y, Deng G, Tanaka Y, Dahiya R (2011). Regulatory role of mir-203 in prostate cancer progression and metastasis. Clin Cancer Res.

[24] Sonkoly E, Loven J, Xu N, Meisgen F, Wei T, Brodin P, Jaks V, Kasper M, Shimokawa T, Harada M, Heilborn J, Hedblad MA, Hippe A, Grander D, Homey B, Zaphiropoulos PG, Arsenian-Henriksson M, Stahle M, Pivarcsi A (2012). Microrna-203 functions as a tumor suppressor in basal cell carcinoma. Oncogenesis.

[25] Linardou H, Dahabreh IJ, Kanaloupiti D, Siannis F, Bafaloukos D, Kosmidis P, Papadimitriou CA, Murray S (2008). Assessment of somatic k-ras mutations as a mechanism associated with resistance to egfr-targeted agents: A systematic review and meta-analysis of studies in advanced non-small-cell lung cancer and metastatic colorectal cancer. Lancet Oncol.

[26] Hanahan D, Weinberg RA (2000). The hallmarks of cancer. Cell.

[27] Letai AG (2008). Diagnosing and exploiting cancer's addiction to blocks in apoptosis. Nat Rev Cancer.

[28] Sentman CL, Shutter JR, Hockenbery D, Kanagawa O, Korsmeyer SJ (1991). Bcl-2 inhibits multiple forms of apoptosis but not negative selection in thymocytes. Cell.

[29] Miyashita T, Reed JC (1992). Bcl-2 gene transfer increases relative resistance of s49.1 and wehi7.2 lymphoid cells to cell death and DNA fragmentation induced by glucocorticoids and multiple chemotherapeutic drugs. Cancer Res.

[30] Carter BS, Epstein JI, Isaacs WB (1990). Ras gene mutations in human prostate cancer. Cancer Res.

[31] Mulholland DJ, Kobayashi N, Ruscetti M, Zhi A, Tran LM, Huang J, Gleave M, Wu H (2012). Pten loss and ras/mapk activation cooperate to promote emt and metastasis initiated from prostate cancer stem/progenitor cells. Cancer Res.

[32] Taylor BS, Schultz N, Hieronymus H, Gopalan A, Xiao Y, Carver BS, Arora VK, Kaushik P, Cerami E, Reva B, Antipin Y, Mitsiades N, Landers T, Dolgalev I, Major JE, Wilson M, Socci ND, Lash AE, Heguy A, Eastham JA, Scher HI, Reuter VE, Scardino PT, Sander C, Sawyers CL, Gerald WL (2010). Integrative genomic profiling of human prostate cancer. Cancer Cell.

[33] Gioeli D, Mandell JW, Petroni GR, Frierson HF, Weber MJ (1999). Activation of mitogen-activated protein kinase associated with prostate cancer progression. Cancer Res.

[34] Malik SN, Brattain M, Ghosh PM, Troyer DA, Prihoda T, Bedolla R, Kreisberg JI (2002). Immunohistochemical demonstration of phospho-akt in high gleason grade prostate cancer. Clin Cancer Res.

[35] Jeong JH, Wang Z, Guimaraes AS, Ouyang X, Figueiredo JL, Ding Z, Jiang S, Guney I, Kang GH, Shin E, Hahn WC, Loda MF, Abate-Shen C, Weissleder R, Chin L (2008). Braf activation initiates but does not maintain invasive prostate adenocarcinoma. PLoS One.

[36] Min J, Zaslavsky A, Fedele G, McLaughlin SK, Reczek EE, De Raedt T, Guney I, Strochlic DE, Macconaill LE, Beroukhim R, Bronson RT, Ryeom S, Hahn WC, Loda M, Cichowski K (2010). An oncogene-tumor suppressor cascade drives metastatic prostate cancer by coordinately activating ras and nuclear factor-kappab. Nat Med.

[37] Yin J, Pollock C, Tracy K, Chock M, Martin P, Oberst M, Kelly K (2007). Activation of the ralgef/ral pathway promotes prostate cancer metastasis to bone. Mol Cell Biol.

[38] Yin JJ, Zhang L, Munasinghe J, Linnoila RI, Kelly K (2010). Cediranib/azd2171 inhibits bone and brain metastasis in a preclinical model of advanced prostate cancer. Cancer Res.

[39] Schwartz YB, Pirrotta V (2008). Polycomb complexes and epigenetic states. Curr Opin Cell Biol.

[40] Margueron R, Reinberg D (2011). The polycomb complex prc2 and its mark in life. Nature.

[41] Herranz N, Pasini D, Diaz VM, Franci C, Gutierrez A, Dave N, Escriva M, Hernandez-Munoz I, Di Croce L, Helin K, Garcia de Herreros A, Peiro S (2008). Polycomb complex 2 is required for e-cadherin repression by the snail1 transcription factor. Mol Cell Biol.

[42] Wellner U, Schubert J, Burk UC, Schmalhofer O, Zhu F, Sonntag A, Waldvogel B, Vannier C, Darling D, zur Hausen A, Brunton VG, Morton J, Sansom O, Schuler J, Stemmler MP, Herzberger C, Hopt U, Keck T, Brabletz S, Brabletz T (2009). The emt-activator zeb1 promotes tumorigenicity by repressing stemness-inhibiting micrornas. Nat Cell Biol.

[43] Cao Q, Mani RS, Ateeq B, Dhanasekaran SM, Asangani IA, Prensner JR, Kim JH, Brenner JC, Jing X, Cao X, Wang R, Li Y, Dahiya A, Wang L, Pandhi M, Lonigro RJ, Wu YM, Tomlins SA, Palanisamy N, Qin Z, Yu J, Maher CA, Varambally S, Chinnaiyan AM (2011). Coordinated regulation of polycomb group complexes through micrornas in cancer. Cancer Cell.

[44] Ru P, Steele R, Hsueh EC, Ray RB (2011). Anti-mir-203 upregulates socs3 expression in breast cancer cells and enhances cisplatin chemosensitivity. Genes Cancer.

[45] Zhang C, Kallakury BV, Ross JS, Mewani RR, Sheehan CE, Sakabe I, Luta G, Kumar D, Yadavalli S, Starr J, Sreenath TL, Srivastava S, Pollard HB, Eidelman O, Srivastava M, Kasid UN (2013). The significance of tnfaip8 in prostate cancer response to radiation and docetaxel and disease recurrence. Int J Cancer.

[46] Morris EJ, Michaud WA, Ji JY, Moon NS, Rocco JW, Dyson NJ (2006). Functional identification of api5 as a suppressor of e2f-dependent apoptosis in vivo. PLoS Genet.

[47] Chen D, Chen Y, Forrest D, Bremner R (2013). E2f2 induces cone photoreceptor apoptosis independent of e2f1 and e2f3. Cell Death Differ.

[48] Schlomm T, Kirstein P, Iwers L, Daniel B, Steuber T, Walz J, Chun FH, Haese A, Kollermann J, Graefen M, Huland H, Sauter G, Simon R, Erbersdobler A (2007). Clinical significance of epidermal growth factor receptor protein overexpression and gene copy number gains in prostate cancer. Clin Cancer Res.

[49] Guérin O, Fischel JL, Ferrero J-M, Bozec A, Milano G (2010). Egfr targeting in hormone-refractory prostate cancer: Current appraisal and prospects for treatment. Pharmaceuticals.

[50] Zhau HE, Wan DS, Zhou J, Miller GJ, von Eschenbach AC (1992). Expression of c-erb b-2/neu proto-oncogene in human prostatic cancer tissues and cell lines. Mol Carcinog.

[51] Pylayeva-Gupta Y, Grabocka E, Bar-Sagi D (2011). Ras oncogenes: Weaving a tumorigenic web. Nat Rev Cancer.

[52] Cho NY, Choi M, Kim BH, Cho YM, Moon KC, Kang GH (2006). Braf and kras mutations in prostatic adenocarcinoma. Int J Cancer.

[53] Shiraishi T, Muneyuki T, Fukutome K, Ito H, Kotake T, Watanabe M, Yatani R (1998). Mutations of ras genes are relatively frequent in japanese prostate cancers: Pointing to genetic differences between populations. Anticancer Res.

[54] Konishi N, Hiasa Y, Tsuzuki T, Tao M, Enomoto T, Miller GJ (1997). Comparison of ras activation in prostate carcinoma in japanese and american men. Prostate.

[55] Ren G, Liu X, Mao X, Zhang Y, Stankiewicz E, Hylands L, Song R, Berney DM, Clark J, Cooper C, Lu YJ (2012). Identification of frequent braf copy number gain and alterations of raf genes in chinese prostate cancer. Genes Chromosomes Cancer.

[56] Pergolizzi RG, Kreis W, Rottach C, Susin M, Broome JD (1993). Mutational status of codons 12 and 13 of the n- and k-ras genes in tissue and cell lines derived from primary and metastatic prostate carcinomas. Cancer Invest.

[57] Liu YN, Abou-Kheir W, Yin JJ, Fang L, Hynes P, Casey O, Hu D, Wan Y, Seng V, Sheppard-Tillman H, Martin P, Kelly K (2012). Critical and reciprocal regulation of klf4 and slug in transforming growth factor beta-initiated prostate cancer epithelial-mesenchymal transition. Mol Cell Biol.

[58] Liu YN, Yin JJ, Abou-Kheir W, Hynes PG, Casey OM, Fang L, Yi M, Stephens RM, Seng V, Sheppard-Tillman H, Martin P, Kelly K (2013). Mir-1 and mir-200 inhibit emt via slug-dependent and tumorigenesis via slug-independent mechanisms. Oncogene.

