# Correction: Loss of EGFR signaling-regulated miR-203 promotes prostate cancer bone metastasis and tyrosine kinase inhibitors resistance

**DOI:** 10.18632/oncotarget.26020

**Published:** 2018-08-17

**Authors:** Man Kit Siu, Wassim Abou-Kheir, Juan Juan Yin, Yung-Sheng Chang, Ben Barrett, Florent Suau, Orla Casey, Wei-Yu Chen, Lei Fang, Paul Hynes, Yao-Yu Hsieh, Yen-Nien Liu, Jiaoti Huang, Kathleen Kelly

**Affiliations:** ^1^ Graduate Institute of Cancer Biology and Drug Discovery, College of Medical Science and Technology, Taipei Medical University, Taipei, Taiwan; ^2^ Department of Anesthesiology, Wan Fang Hospital, Taipei Medical University, Taipei, Taiwan; ^3^ Department of Anatomy, Cell Biology and Physiological Sciences Faculty of Medicine, American University of Beirut, Beirut, Lebanon; ^4^ Cell and Cancer Biology Branch, National Cancer Institute, National Institutes of Health, Bethesda, MD, USA; ^5^ Department of Pathology, Wan Fang Hospital, College of Medicine, Taipei Medical University, Taipei, Taiwan; ^6^ Division of Hematology and Oncology, Shuang Ho Hospital, Taipei Medical University, New Taipei City, Taiwan; ^7^ Center of Excellence for Cancer Research, Wan Fang Hospital, College of Medicine, Taipei Medical University, Taipei, Taiwan; ^8^ Department of Pathology and Laboratory Medicine, University of California, Los Angeles, CA, USA; ^9^ Department of Internal Medicine, Division of Hematology and Oncology, School of Medicine, College of Medicine, Taipei Medical University, Taipei, Taiwan

**This article has been corrected:** The author affiliation is given below:

**Yao-Yu Hsieh^1,6,9^**

^9^ Department of Internal Medicine, Division of Hematology and Oncology, School of Medicine, College of Medicine, Taipei Medical University, Taipei, Taiwan

Original article: Oncotarget. 2014; 5:3770-3784. https://doi.org/10.18632/oncotarget.1994

