# Supplementary material for: Loss of EGFR signaling-regulated miR-203 promotes prostate cancer bone metastasis and tyrosine kinase inhibitors resistance
Source: Oncotarget. 2014 May 20;5(11):3770–84. doi: 10.18632/oncotarget.1994 (PMC4116519; doi:10.18632/oncotarget.1994)
Supplement: Supplementary file 1 [file oncotarget-05-3770-s001.pdf]

Loss of EGFR signaling regulated miR-203 promotes prostate cancer bone metastasis and tyrosine kinase inhibitors resistance

SUPPLEMENTAL MATERIALS AND METHODS

Reagents and Constructs

SMARTpool *SUZ12* siRNA were from Thermo Scientific (Thermo Scientific, CO). All siRNA sequences are listed in Supplemental Tables (Table S4). The human *SUZ12* miR-203 response element 3'UTR reporter was constructed using the psiHECKTM-2 vector (Promega, WI). The position of E boxes located upstream of human primary *hsa-mir-203* on chromosome 14 at GRCh37 are as follows: E1: 14:104583212, E2: 14: 104583081, E33: 14: 104582721, E4: 14: 104581917, E5: 14: 104581688, E6: 14: 104581404, E7: 14: 104581349, E8: 14: 104581295. E boxes mutations were made using the Site-Directed Mutagenesis System kit (Invitrogen, CA). The human *SUZ12* and *SNAI1* full length cDNA expression vectors were constructed using pCDH-CMV-MCS-EF1-Pure vector (System Biosciences, CA). All primers used for these constructs are listed in Supplemental Tables (Table S1). All constructs were verified by DNA sequence analysis.

Real-time RT-PCR

Total RNA was isolated using mirVana PARIS RNA isolation system (Ambion, TX). Reverse transcription of cDNA and PCR were performed as described [1, 2]. All reactions were normalized to human *GAPDH* and were run in triplicate using primers listed in Supplemental Tables (Table S2). MicroRNA reverse transcription and PCR reactions were performed using TaqMan MicroRNA Assay kits (Applied Biosystems, CA). All values were normalized to human *SNORD48* endogenous control and were run in triplicate (Applied Biosystems, CA). The clinical

samples from 25 with independent prostate tumors used in qRT-PCR analyses were collected form Wan Fang Hospital, Taipei Medical University. RNA was extracted from dissected tissue containing greater than 70% tumor cell content.

MicroRNA Luciferase Assay

RasB1 cells were transfected with 1μg of human *SUZ12, AREG, EREG,* and *TGFA* 3'UTR reporter and 1μg of precursor miR's encoding empty vector or miR-203 precursor. The psiCHECK-2 vectors contain both firefly and Renilla luciferase reporters. Cell extracts were prepared 24 hours after EGF (10μM) or CI1033 (10nM) treatment and the luciferase and Renilla activities were measured using the Dual Luciferase Reporter Assay System (Promega, WI). Renilla luciferase activities were calculated as the mean ± SEM after normalization to firefly luciferase activities. Three independent experiments were done in triplicate.

FACS Analysis

Promoter functional analysis using FACS and relative MFI (median fluorescent intensity) value was measured as described [1, 2]. Cells were treated with or without EGF (10μM), and CI1033 (10nM) for 48 hours. The MFI (median fluorescent intensity) value for RFP was measured by FACS using FACSDiva software and normalized to the value of the vehicle. Promoter functional analysis using FACS and relative MFI value was measured as described [3]. Mean fluorescent intensities were determined from the first peak of fluorescence (which occurs in >80% of the cells since conditions are used to optimize single lentivirus incorporation) and represent single copy integration.

Chromatin Immunoprecipitation (ChIP)

ChIP assays were performed using the EZ magna ChIP A kit (Millipore, CA) with a modified protocol. Cells were treated with or without EGF (10µM) for 24 hours. Cultured cells ($1X10^7$) were cross-linked with 1% formaldehyde at RT for 15 minutes. The fixation was quenched with glycine, and cells were washed twice with cold PBS containing complete protease inhibitor (Roche, CA). Cell pellets were resuspended in cell lysis buffer and incubated on ice for 15 minutes. Nuclei were collected by centrifugation at 10,000 rpm at 4°C for 10 minutes and resuspended in nuclei lysis buffer. Chromatin was sheared using a sonicator (Branson Sonifier 250, Teltow, Germany) with a microtip in 20-second burst followed by 1 minutes of cooling on ice for a total sonication time of 5 minutes per sample. The procedure results in DNA fragment sizes of 100-300 bp. Sheared chromatin was divided to perform immunoprecipitation with rabbit IgG antibody (Santa Cruz Biotechnology, CA) or primary antibody at 4°C overnight. Immunoprecipitation, washing, elution, reverse cross-linking, and DNA purification steps were performed according to Millipore's protocol. Quantitative PCR was performed in triplicate with 2µl of eluted chromatin. ChIP antibodies and PCR primers are listed in Supplemental Tables (Table S5).

Immunohistochemistry (IHC) staining

Immunohistochemistry (IHC) was performed using EREG antibodies from R&D (R&D Systems, MN) at 1:60 dilution. In general, unstained sections were deparaffinized and rehydrated. Antigen retrieval was performed using Target Antigen Retrieval Solution (DAKO, CA) and autoclave for 10 minutes. For IHC, endogenous peroxidase was blocked using a 3% hydrogen peroxide solution. All sections were blocked with Cyto Q Background Buster Reagent (Innovex BioSciences, CA). Primary antibodies were incubated overnight at 4°C in Antibody Diluent with Background

Reducing Components (DAKO, CA). Secondary antibody, 1:250 HRP labeled anti mouse/rabbit (Vector laboratories, CA), incubation was performed at room temperature for 30 minutes and bound peroxidase detected using the ABC Peroxidase Kit (Vector laboratories, CA) and DAB (DAKO, CA). All IHC slides were counterstained with hematoxylin. For histomorphometric analysis of tissue sections, microscopic images were collected under 200X magnification using an Axioplan microscopy system (Zeiss, Thornwood, New York).

Clinical outcome and correlation analyses using human data sets

We used mRNA expression data from a public human prostate cancer data set [4, 5]. The study was conducted under MSKCC Institutional Review Board approval on 28 normal, 151 primary, and 19 metastatic samples. The expression data (and resulting z-scores) were log2 normalized. Additionally, microRNA expression was determined for 113 tumors and 28 matched normal samples with Agilent microRNA V2 arrays. KRAS (Broad Institute), metastasis [6], and EGFR signaling [7] responsive gene signatures were used to determine correlations with miR-203 levels. Gene sets were scored by summing the expression Z score per tumor within the cohort. Tumors were mean stratified by miR-203 expression and the mean Z scores were determined in each group.

Statistical Analysis

In vivo animal results and clinical outcome analysis are expressed as plots showing the median and box boundaries extending between 25th to 75th percentiles, with whiskers down to the minimum and up to the maximum value. All in vitro data were presented as means ± S.E.M. Statistical calculations were performed with GraphPad Prism (GraphPad Software, Inc.) analysis tools. Differences between individual groups were analyzed by one way or two way ANOVA test.

Bonferroni's post test was used for comparisons among 3 or more groups. Log-rank test was used for survival curve analysis. P values less than 0.05 were considered statistically significant.

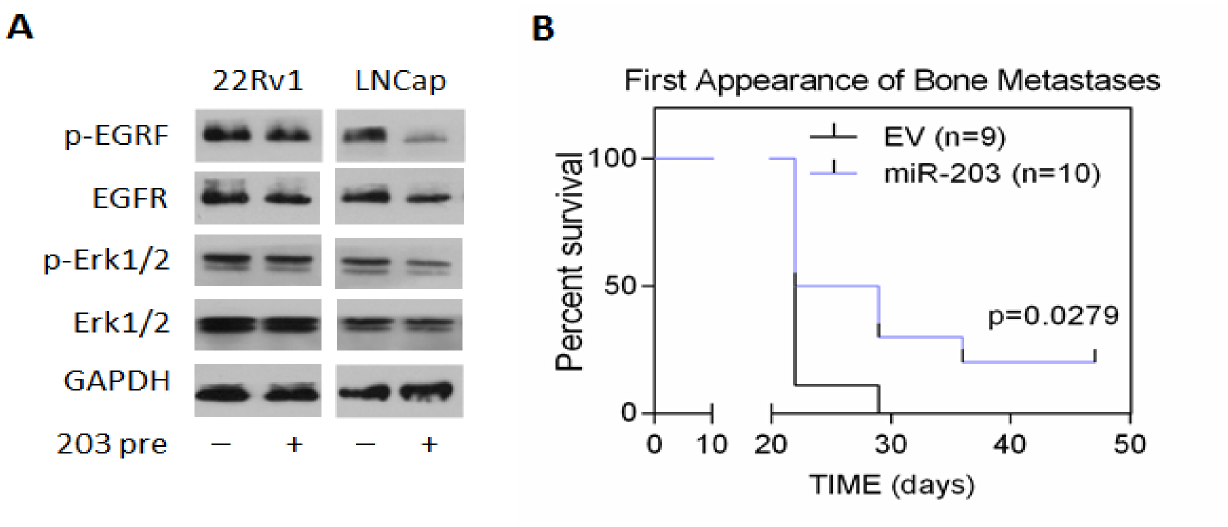

Figure S1: miR-203 alters the expression of EGFR/ERK signaling in LNCap cells and increase in survival rate in RasB1 cells injection mice. (A) Representative Western Blot analysis of p-EGFR, EGFR, p-ERK1/2, and ERK1/2 in two AR positive cell lines 22Rv1 and LNCap transiently transfected with control and miR-203 microRNA precursor. (B) RasB1 cells over-expressing miR-203 precursor showed a significant increase in survival rate compared to empty vector following intracardiac injection of mice with RasB1 cells expressing empty vector (n=9) or miR-203 (n=10) precursor. Kaplen-meier plot showing percent survival over time where first appearance of long bone or brain metastasis was determined by bioluminescence imaging.

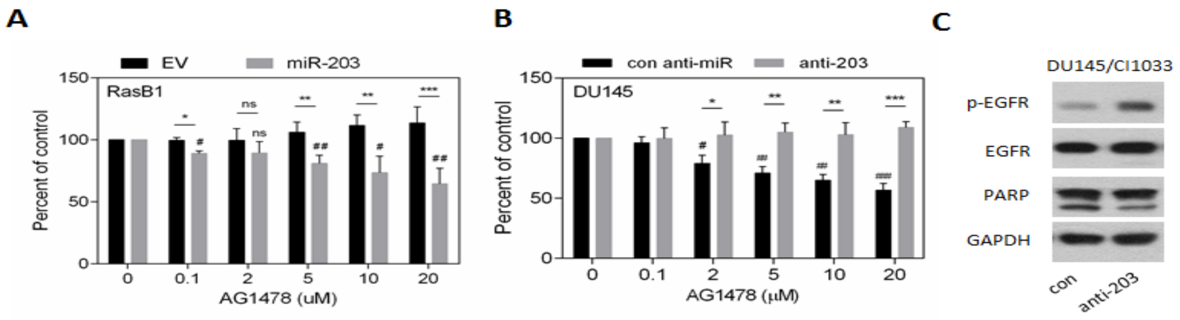

Figure S2: miR-203 overexpression induces cell death of TKI-resistant RAS-activated prostate cancer cells. (A) RasB1 with empty vector (EV) or miR-203 precursor cells were treated with increasing concentrations of AG1478. *: vs. EV. (B) DU145 with control anti-miR or anti-miR-203 inhibitor cells were treated with increasing concentrations of AG1478. Cell viability, relative to untreated controls, was measured at 24 hours. Each data point represents the mean± s.d. of six wells. *: vs. control anti-miR, #: vs. 0 nM. *p<0.05. **p<0.01, ***p<0.001. (C) Representative Western Blot analysis of p-EGFR, EGFR, and PARP in DU145 cell lines transiently transfected with control anti-miR or anti-miR-203 inhibitor.

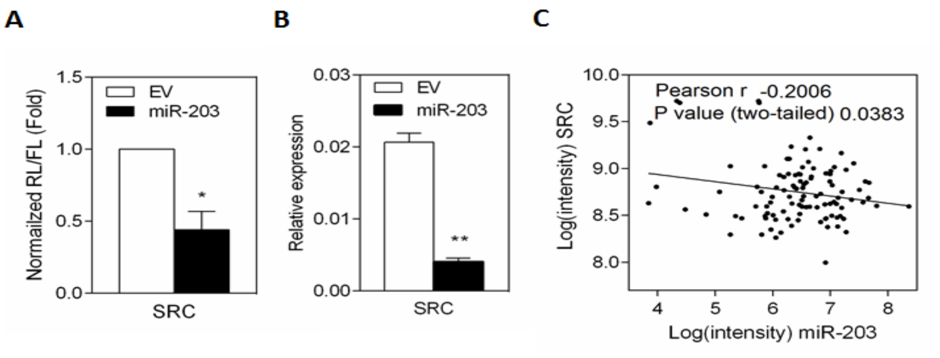

Figure S3: miR-203 overexpression induces repression of SRC by directly targeting the 3'UTR of *SRC*. (A) The normalized reporter activity of *SRC* 3'UTR containing miR-203 target reporter in RasB1 cells with miR-203 precursor or empty vector (EV). Renilla/luciferase activities were measured 48 hours after transfection. Data represent means ± SEM of separate transfections, n=3. *: vs. EV. (B) qRT-PCR analysis of *SRC* level in RasB1 cells expressing miR-203 precursor or empty vector. Relative mRNA expression was normalized to *GAPDH*. Data represent means ± SEM of separate transfections, n=3. *: vs. EV. *p<0.05, **p<0.01. **(C)** Pearson anti-correlation coefficient of mean miR-203 to mean *SRC* mRNA expression in primary and metastasis prostate samples (n=111). Significance determined by Gaussian population (Pearson) and two-tailed test.

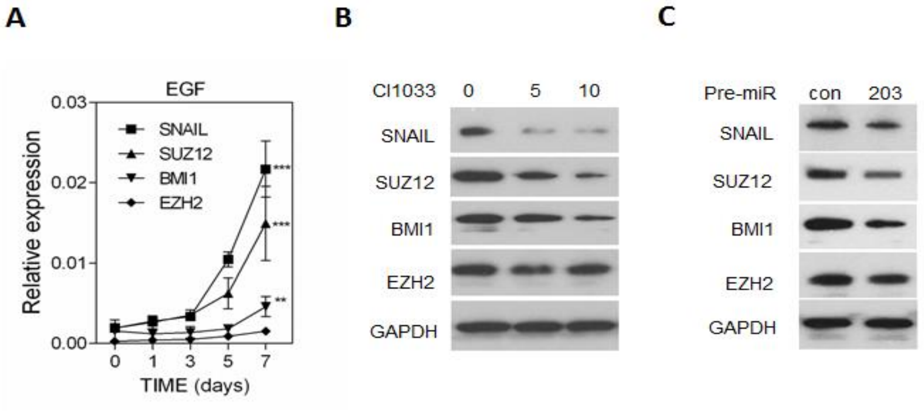

Figure S4: Activated EGFR signaling induced SNAIL and SUZ12 is regulated by miR-203 in RasB1 cells. (A) qRT-PCR of *SNAIL* and PRCs levels determined in RasB1 cells after EGF treatment. Data represent means ± SEM, n=3. *: responsiveness to EGF. (B) Representative Western Blot analysis of SNAIL, SUZ12, BMI1, EZH2, and GAPDH in RasB1 cells after CI1033 treatment for 24 hours. (C) Representative Western Blot analysis of SNAIL, SUZ12, BMI1, EZH2, and GAPDH in RasB1 cells stably transfected with control (EV) or miR-203 precursor.

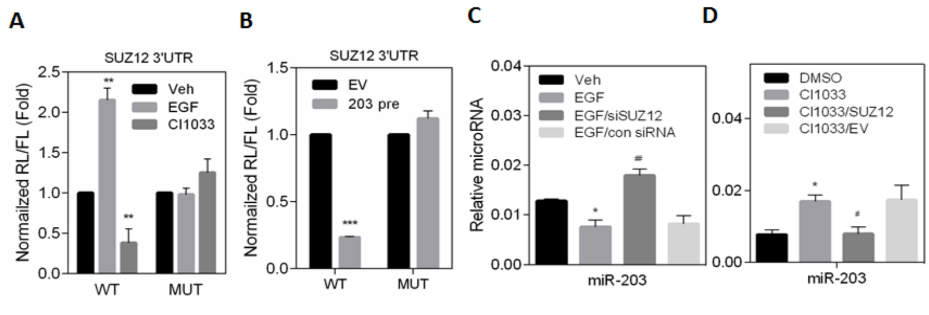

Figure S5: miR-203 overexpression induces repression of SUZ12 by directly targeting the 3'UTR of *SUZ12*. (A and B) The normalized reporter activity of *SUZ12* 3'UTR containing wild type or mutated miR-203 target reporter in RasB1 cells with EGF or CI1033 treatment (A) and transient expression of miR-203 precursor or empty vector (EV) (B). Renilla/luciferase activities were measured 48 hours after transfection. Data represent means ± SEM of separate transfections, n=3. *: vs. vehicle or EV. (C) qRT-PCR analysis of miR-203 level in RasB1 cells with SMARTpool *SUZ12* or control siRNA introduction after EGF treatment. *: vs. vehicle, #: vs. control siRNA. (D) qRT-PCR analysis of miR-203 level in RasB1 cells expressing SUZ12 or empty vector (EV) after being treated with 10nM CI1033 or DMSO for 24 hours. *: vs. DMSO, #: vs. EV. *p<0.05, **p<0.01, ***p<0.001.

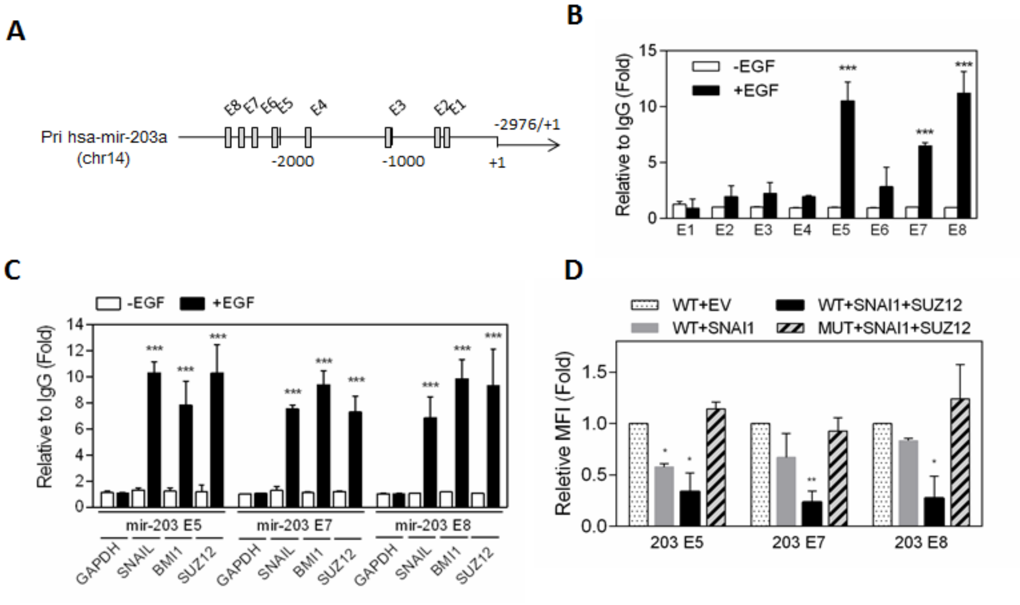

Figure S6: *Pri-mir-203a* is regulated through a mutually inhibitory feed-forward loop by SUZ12 and SNAIL. (A) Schematic of predicted E-boxes in the *hsa-mir-203a* primary miR (pri-mir) stem-loop promoter. (B) qChIP analysis of SNAIL binding to predicted E-boxes in the *pri-mir-203a* promoter region measured in RasB1 cells treated with EGF (10μM) for 24 hours. The binding activity of each protein to each site is given as a percentage of total input then normalized to each IgG. Data are means ± SEM, n=3. *: vs. -EGF. (C) qChIP analysis of SNAIL, BMI1, and SUZ12 to the indicated elements in the *pri-mir-203a* promoter region in RasB1 cells following EGF treatment for 24 hours. Data represent means ± SEM, n=3. *: vs. -EGF. (D) RasB1 cells were transiently co-transfected with wild type or E-box mutated *pri-mir-203a* RFP reporters with SUZ12 or SNAIL expression vectors for 48 hours. The MFI was measured by FACS and normalized to the value of the empty vector. Data represent means ± SEM, n=3. *: vs. empty vector (EV). *p<0.05, **p<0.01, ***p<0.001.

**Table S1**	
3'UTR reporter constructs primer sequence	
hSUZ12/psi-2 3'UTR (PmeI) F	attcgtttaaacTGTTATGGACAAACACTGAAATTACA
hSUZ12/psi-2 3'UTR (NotI) R	attcgtttaaacCAACATATTTGATGACATTTGTTGAGT
hSUZ12/psi-2 225M F	GCATGAAAAGCAATAGGGAAAAGTATTTTTAA
hSUZ12/psi-2 225M R	TTGTAAGACATGAAATTCGTACTTTTCGTTA
hAREG/psi-2 3'UTR (XhoI) F	atcgctcgagCTGAAGATAAAATTACAGGATAT
hAREG/psi-2 3'UTR (PmeI) R	agagcggccgcTGAGCTGTAAAATAAATACACTT
hAREG/psi-2 908M F	AAATCCATGTAATGCAGAAGGGAAAAATTTCTGC
hAREG/psi-2 908M R	TGTCTTTCTTCTTTTTAGGTACATTACGTCT
hEREG/psi-2 3'UTR (XhoI) F	atcgctcgagGGGATAACAGTGTGCCTGGT
hEREG/psi-2 3'UTR (PmeI) R	agagcggccgcTTTTGAATGCAAGATGTTCAATG
hEREG/psi-2 734M F	CTTTTATTTAAGTAGTGGGAAGGGAATAGCTTCACA
hEREG/psi-2 734M R	TAAAAGACATTAGAAAATAAATTCATCACCC
hTGFA/psi-2 3'UTR (XhoI) F	atcgctcgagCAGAGGAGGAGTTTGGCCA
hTGFA/psi-2 3'UTR (PmeI) R	agagcggccgcAGATTCATTCCTTCATCCTTCCA
hTGFA/psi-2 2337M F	GTTAGCTGCAAAAATACTCAAGGGAAAGTTAAAAAT
hTGFA/psi-2 2337M R	ACCATCAGAAACCAATCGACGTTTTTATGAG
hTGFA/psi-2 1072M F	ATTAGCCACGAGCCATAGGAAGGGAAGGCCAAATCC
hTGFA/psi-2 1072M R	ATCTTTATCTTGTAATCGGTGCTCGGTATCC
hTGFA/psi-2 219M F	AAGAAATCAGCGGACCACGAGGGAAAGACTTGTTAA
hTGFA/psi-2 219M R	ACACTGGATCTCTTCTTTAGTCGCCTGGTGC
Has-mir-203 promoter reporter primer sequence	
Has-miR-203E5 P1	CTCTCGTCAGTTCCCTTGGA
Has-miR-203E5 P2	CAGTCCTGGGCAGACTCTTC
Has-miR-203E5 P3	GAGGCAGCAGAGACCGCTCTCGTCAGTTCCCTTGGA
Has-miR-203E5 P4	CGAACAGAGAGAGACCGCAGTCCTGGGCAGACTCTT
Has-miR-203E5M P1	CACGGGAGCCAATTGGGCGAGG
Has-miR-203E7 P1	CCGCTCACACCTGGTAATTT
Has-miR-203E7 P2	GGCTATAGGAACCAAGGCCT
Has-miR-203E7 P3	GAGGCAGCAGAGACCGCCGCTCACACCTGGTAATTT
Has-miR-203E7 P4	CGAACAGAGAGAGACCGGGCTATAGGAACCAAGGC
Has-miR-203E7M P1	CGCCGCTCACAATTGGGTAATTTC
Has-miR-203E8 P1	GCCTTGTCTCTTGCTGTGG
Has-miR-203E8 P2	ACCTGGCTGTGCCTCATT
Has-miR-203E8 P3	GAGGCAGCAGAGACCGGCCTTGTCTCTTGCTGTGG
Has-miR-203E8 P4	CGAACAGAGAGAGACCGACCTGGCTGTGCCTCATT
Has-miR-203E8M P1	TGAGGCACAGCCAATTGCCCAGTGC
Full length cDNA expression vector primer sequence	
pCDH/hSNAIL cDNA NheI F	gctagcatgccgcgctctttcctcgt
pCDH/hSNAIL cDNA XbaI R	tctagatcagcggggagatggtgagc
pCDH/hSUZ12 cDNA NheI F	gctagcatggcgcctcagaagcacggc
pCDH/hSUZ12 cDNA XbaI R	tctagatcagagtttttgtttttgc

**Table S2**		
qRT-PCR primer sequence	5'-3'	
hGapdh F	GGACTCATGACCACAGTCCA	
hGapdh R	CCAGTAGAGGCAGGGATGAT	
hBmi1 F	CTTTCATTGTCTTTTCCGCC	
hBmi1 R	CAGGTGGGGATTTAGCTCAG	
hSuz12 F	TGAAGTAGCCATGCAGGAAA	
hSuz12 R	TCCAACGAAGAGTGAACTGC	
hSnail F	CTAGGCCCTGGCTGCTAC	
hSnail R	GACATCTGAGTGGGTCTGGA	
hEzh2 F	AGCGGAAGAACACAGAAACA	
hEzh2 R	GTGAGAGCAGCAGCAAACTC	
hAREG	TGATCCTCACAGCTGTTGCT	
hAREG	CATTTCCATTCTCTTGTCGAAG	
hEREG F	AATGGCTATTGTTTGCATGG	
hEREG R	CTTAAAGGTTGGTGGACGGT	
hTGFA F	CTGATACACTGCTGCCAGGT	
hTGFA R	CTCCTCTGGGCTCTTCAGAC	
hAPI5 F	CAGGCTGCAGTACTTTGCAC	
hAPI5 R	TTTAAGGCCTCACCCGTTT	
hBIRC2 F	TGGCATCAGATGATTTGTCA	
hBIRC2 R	TCCAGGATAGGAAGCACACA	
hTRIAP1 F	GCACCGACCTCTTCAAGC	
hTRIAP1 R	CCATGAACTCCAGTCCTTCA	
hTNFAIP8 F	TAGTGAGGTGCTGGATGAGC	
hTNFAIP8 R	TGATGATCTTCTCTGCCTCCT	

**Table S3**					
WB antibodies					
Primary antibody	Source	Dilution	Secondary antibody	Source	Dilution
p-EGFR	Cell Signaling (#3777)	1/1000	anti rabbit IgG	Thermo Scientific	1/2000
EGFR	Cell Signaling (#4267)	1/1000	anti rabbit IgG	Thermo Scientific	1/2000
p-ERK1/2	Cell Signaling (#4376)	1/2000	anti rabbit IgG	Thermo Scientific	1/2000
ERK1/2	Cell Signaling (#9102)	1/1000	anti rabbit IgG	Thermo Scientific	1/2000
Bmi1, clone F6	Millipore (#05-637)	1/1000	anti mouse IgG	Thermo Scientific	1/2000
Suz12 (D39F6)	Cell Signaling (#3737)	1/1000	anti rabbit IgG	Thermo Scientific	1/2000
SNAIL	Cell Signaling (#3879)	1/2000	anti rabbit IgG	Thermo Scientific	1/2000
GAPDH	Novus (NB300-221)	1/4000	anti mouse IgG	Thermo Scientific	1/2000

**Table S4**		
siRNA sequence		
Human SUZ12 siRNA SMART pool	GAACACCUAUCACACAUAU	
	GCAACAAACUGAAGCAAGA	
	GAACAGCAAAGAACAUAUA	
	GAAUUUAUGUCGAAACUUC	

**Table S5**		
ChIP antibodies		
Primary antibody	Source	Dilution
SNAIL	Cell Signaling (#3879)	1/50
Bmi1, clone F6	Millipore (#05-637)	1/50
Suz12 (D39F6)	Cell Signaling (#3737)	1/50
GAPDH	Novus (NB300-221)	1/50
Rabbit IgG	Santa Cruz (sc-2027)	1/50
ChIP primers		
Site	5'-3'	
203ChE1 F	GCCCAGACGAGACGGTTC	
203ChE1 R	GACTGATCCTCCACGGCC	
203ChE2 F	GTCCTCACCTGTTCCGGC	
203ChE2 R	GAACCGTCTCGTCTGGGC	
203ChE3 F	CTCAGCTGCCATGTTCTGC	
203ChE3 R	ACCCACTTAGTCACCTGGAG	
203ChE4 F	CCAACATGGCGAAACTCTGT	
203ChE4 R	CACCTCAGCCTCCCAAGTAG	
203ChE5 F	CTCTCGTCAGTTCCCTTGGA	
203ChE5 R	CAGTCCTGGGCAGACTCTTC	
203ChE6 F	AGGCCTTGGTTCCTATAGCC	
203ChE6 R	GTCGTGATGGAGAGGAGATGT	
203ChE7 F	CCGCTCACACCTGGTAATTT	
203ChE7 R	GGCTATAGGAACCAAGGCCT	
203ChE8 F	GCCTTGTCTCTTGCTGTGG	
203ChE8 R	ACCTGGCTGTGCCTCATT	

REFERENCES

1 Liu YN, Abou-Kheir W, Yin JJ, Fang L, Hynes P, Casey O, Hu D, Wan Y, Seng V, Sheppard-Tillman H, Martin P, Kelly K: Critical and reciprocal regulation of klf4 and slug in transforming growth factor beta-initiated prostate cancer epithelial-mesenchymal transition. Mol Cell Biol 2012;32:941-953.

2 Liu YN, Yin JJ, Abou-Kheir W, Hynes PG, Casey OM, Fang L, Yi M, Stephens RM, Seng V, Sheppard-Tillman H, Martin P, Kelly K: Mir-1 and mir-200 inhibit emt via slug-dependent and tumorigenesis via slug-independent mechanisms. Oncogene 2013;32:296-306.

3 Day CP, Carter J, Bonomi C, Esposito D, Crise B, Ortiz-Conde B, Hollingshead M, Merlino G: Lentivirus-mediated bifunctional cell labeling for in vivo melanoma study. Pigment Cell Melanoma Res 2009;22:283-295.

4 Mulholland DJ, Kobayashi N, Ruscetti M, Zhi A, Tran LM, Huang J, Gleave M, Wu H: Pten loss and ras/mapk activation cooperate to promote emt and metastasis initiated from prostate cancer stem/progenitor cells. Cancer Res 2012;72:1878-1889.

5 Taylor BS, Schultz N, Hieronymus H, Gopalan A, Xiao Y, Carver BS, Arora VK, Kaushik P, Cerami E, Reva B, Antipin Y, Mitsiades N, Landers T, Dolgalev I, Major JE, Wilson M, Socci ND, Lash AE, Heguy A, Eastham JA, Scher HI, Reuter VE, Scardino PT, Sander C, Sawyers CL, Gerald WL: Integrative genomic profiling of human prostate cancer. Cancer Cell 2010;18:11-22.

6 Chandran UR, Ma C, Dhir R, Bisceglia M, Lyons-Weiler M, Liang W, Michalopoulos G, Becich M, Monzon FA: Gene expression profiles of prostate cancer reveal involvement of multiple molecular pathways in the metastatic process. BMC Cancer 2007;7:64.

7 Ma Y, Croxton R, Moorer RL, Jr., Cress WD: Identification of novel e2f1-regulated genes by microarray. Arch Biochem Biophys 2002;399:212-224.
